# Impact of planting dates on yield and resistance of soybean varieties to soybean stem fly (*Melanagromyza sojae*) in Egypt

**DOI:** 10.1038/s41598-025-19034-2

**Published:** 2025-09-18

**Authors:** Eman Ibrahim Abdel-Wahab, Magda Hanna Naroz, Soheir Farouk Abd El-Rahman

**Affiliations:** 1 Food Legumes Research Department, Field Crops Research Institute, Agricultural Research Center (ARC), Postal Box 12619, Giza, Egypt; 2 Economic Entomology and Pesticides Department, Faculty of Agriculture, Cairo University, Postal Box 12622, Giza, Egypt; 3 Plant Protection Research Institute, Agricultural Research Center (ARC), Dokki, Giza, Egypt

**Keywords:** Soybean varieties, Planting dates, Soybean stem fly, Stem anthocyanins content, Leaf total phenols content, Biotechnology, Plant sciences

## Abstract

Late planting dates can significantly reduce soybean yield due to fluctuations in soybean stem fly infestation levels in soybean crops. A two-year study was carried out at ARC, Giza, Egypt during 2020 and 2021 summer seasons to assess varietal performance to the infestation of soybean stem flies during three planting dates. Comparing to planting soybeans in mid-June, planting soybeans in mid-May resulted in increased days to maturity by 6.29% in the first season and 8.04% in the second season, increased seed yield per plot by 12.88% in the first season and 10.94% in the second season, and improved yield attributes such as 100-seed weight and pods per plant. Planting Giza 111 and Giza 35 in mid-May produced 2.78 and 2.75 kg/plot in the first season, and 2.40 and 2.43 kg/plot in the second season, respectively, with minimal stem fly infestation (52.77 and 47.49% damage reduction in the first season, and 40.00 and 39.94% damage reduction in the second season, respectively). Planting Dr-101 in mid-June produced 2.45 and 2.31 kg/plot in the first and second seasons, respectively, with minimal stem fly infestation (20.93 and 12.34% damage reduction in the first and second seasons, respectively). Planting Giza 111 and Giza 35 in mid-May is the best approach to maximize seed yield while reducing damage from soybean stem flies. Alternatively, planting Dr-101 in mid-June could also be a suitable option for achieving high yields with minimal pest infestation.

**Supplementary Information:**

The online version contains supplementary material available at 10.1038/s41598-025-19034-2.

## Introduction

Soybeans (*Glycine max* L.) are a valuable crop used for oil production, industrial derivatives, animal feed, and human consumption^1^. Nearly 80% of the world’s soybean-growing land is concentrated in three major nations: Argentina, Brazil, and the United States^2^. They also noted that Turkey (41.2 c/ha), the United States and Brazil (34.0 c/ha), and Canada and Argentina (30.9 c/ha) and (30.3 c/ha) were the top three countries with the highest yield levels, according to a ranking by the Department of Agriculture (USDA) using statistical data from the U.S. Foreign Agricultural Service.

Soybeans are a crucial summer crop in Egypt, valued for their nutritious oil and as feed for fish, poultry, and cattle. Over the past decade, the cultivation area of soybeans has increased significantly from 8785 hectares with an average yield of 3.23 t/ha in 2011 to 63,461 hectares with an average yield of 2.95 in 2022 (Source: Agriculture Economic sector statistics “AESS”2023, Available at: https://www.agri.gov.eg/library/25)^3^. This growth can be attributed to economic fluctuations worldwide, particularly in the Middle East. Insect infestations, particularly soybean stem flies (*Melanagromyza sojae*), have posed challenges to agricultural development in the region, leading to a decrease in average yield over the past decade to 8.66%. Soybean stem fly (*Melanagromyza sojae*) significantly reduces soybean yields and affects other Fabaceae species^4^. The larvae of soybean stem fly can tunnel up to 70% of the stem length and infest all soybean varieties^5^. Treating soybean stem fly is challenging as it resides inside plant tissues, making it difficult to target with natural predators or insecticides. Soybean stem flies can cause a 20–30% reduction in yield, especially during the early vegetative stage^6^. Yield losses may occur if leaves with smaller seeds wilt or die during the pod-filling process. The peak of the life cycle of soybean stem flies in Egypt occurs during the months of July and August, which coincide with the hottest and humid period of the year. During this time, populations of soybean stem flies are at their highest and can cause significant damage to soybean crops if not effectively managed. Therefore, it is crucial to test the resistance of soybean varieties to infestation by soybean stem flies before planting in June to ensure successful crop production and minimize potential losses. When planted early in the growing season (May), Egyptian varieties showed resistance to insects^7,8^. Previous studies found that the choice of soybean variety and planting date significantly influenced yield. However, further research is needed to address soybean stem fly infestations. By investigating the effects of planting date and soybean variety on infestation, this study aims to fill existing knowledge gaps. Understanding how different varieties and planting times affect susceptibility to infestation can help farmers make informed decisions to reduce yield losses. Future research could focus on developing integrated pest management strategies that combine planting practices and resistant varieties to effectively control soybean stem fly populations.

Maturity group (MG) of soybeans plays a crucial role in avoiding or minimizing infestation by soybean stem fly. Variations in MG account for differences in stem lignin (a phenolic compound) and the pubescence density among soybean varieties^8^. Planting delays beyond late May and early June lead to reduced seed yields^9^. Its sensitivity to photoperiod and temperature makes it a valuable parent for developing widely adaptable soybean varieties. Accordingly, early planting dates and selecting a suitable MG can optimize the number of days for growth before full maturity, leading to an extended seed filling phase and increased soybean seed yield^10^. Soybean variety Giza 111 had the lowest when planted at end of May^11^. They added that planting Giza 111 at beginning of May resulted in the highest seed yield/unit area. Therefore, the study aimed to assess varietal performance to the infestation of soybean stem flies during three planting dates.

## Materials and methods

### Study area

The experiments of the present study were carried out at Giza Agricultural Research Station (Lat. 30°00′30″ N, Long. 31°12′43″ E, 26 m a.s.l), ARC, Giza, Egypt during 2020 and 2021 summer seasons. Eighteen treatments were the combinations between three planting dates (mid-May, beginning of June, and mid-June) and six soybean varieties (Dr-101, Crawford, Giza 21, Giza 22, Giza 35, and Giza 111). The common names, pedigree, growth habit, MG, and origin of these varieties are listed in Table 1. The Food Legumes Research Department at the Field Crops Research Institute, ARC, Egypt, provided the tested soybean varieties. Furrow irrigation was the irrigation system in the region. Soil samples (0–30 cm depth) were analyzed for texture, pH, organic matter, and available nutrients content at the Soils, Water, and Environment Research Institute, ARC, Egypt. The soil analysis revealed that the experimental soil is clay loamy, with percentages of coarse sand (3.22% in the first season, 3.02% in the second season), fine sand (30.59% in the first season, 31.21% in the second season), silt (27.23% in the first season, 29.26% in the second season), and clay (38.96% in the first season, 36.51% in the second season). The pH (paste extract) was measured at 7.89 in the first season and 8.13 in the second season. Organic matter (2.06% in the first season, 2.14% in the second season). The available nutrients in mg/kg were nitrogen (25.35 in the first season, 26.85 in the second season), phosphorus (9.73 in the first season, 10.88 in the second season), and potassium (215 in the first season, 242 in the second season).

**Table 1:** Common names, pedigree, growth habit, MG, and origin of six soybean varieties.

Soybean variety	Pedigree	Growth habit	MG	Origin
Crawford	Williams x Columbus	Indeterminate	IV	USA
Dr-101	Selected from Elgin	Determinate	V	USA
Giza 35	Crawford x Celeste (early)	Indeterminate	III	FCRI
Giza 22	Giza 21 × L86-k73	Indeterminate	IV	FCRI
Giza 21	Crawford x Forrest	Indeterminate	IV	FCRI
Giza 111	Crawford x Celeste (late)	Indeterminate	IV	FCRI

Maturity group (MG): As MG number increases, the lengths of the vegetative and reproductive stages of development are extended. An interval of about 10 days in maturity exists for genotypes classified in the same MG. Early maturity genotypes (III) were adapted to the more northern climatic regions with the maturity designation increasing as you move south. FCRI: Field Crops Research Institute, Giza, Egypt. USA: US Regional Soybean Laboratory at Urbana, Illinois, and Stoneville, Mississippi.

**Fig. 1:**
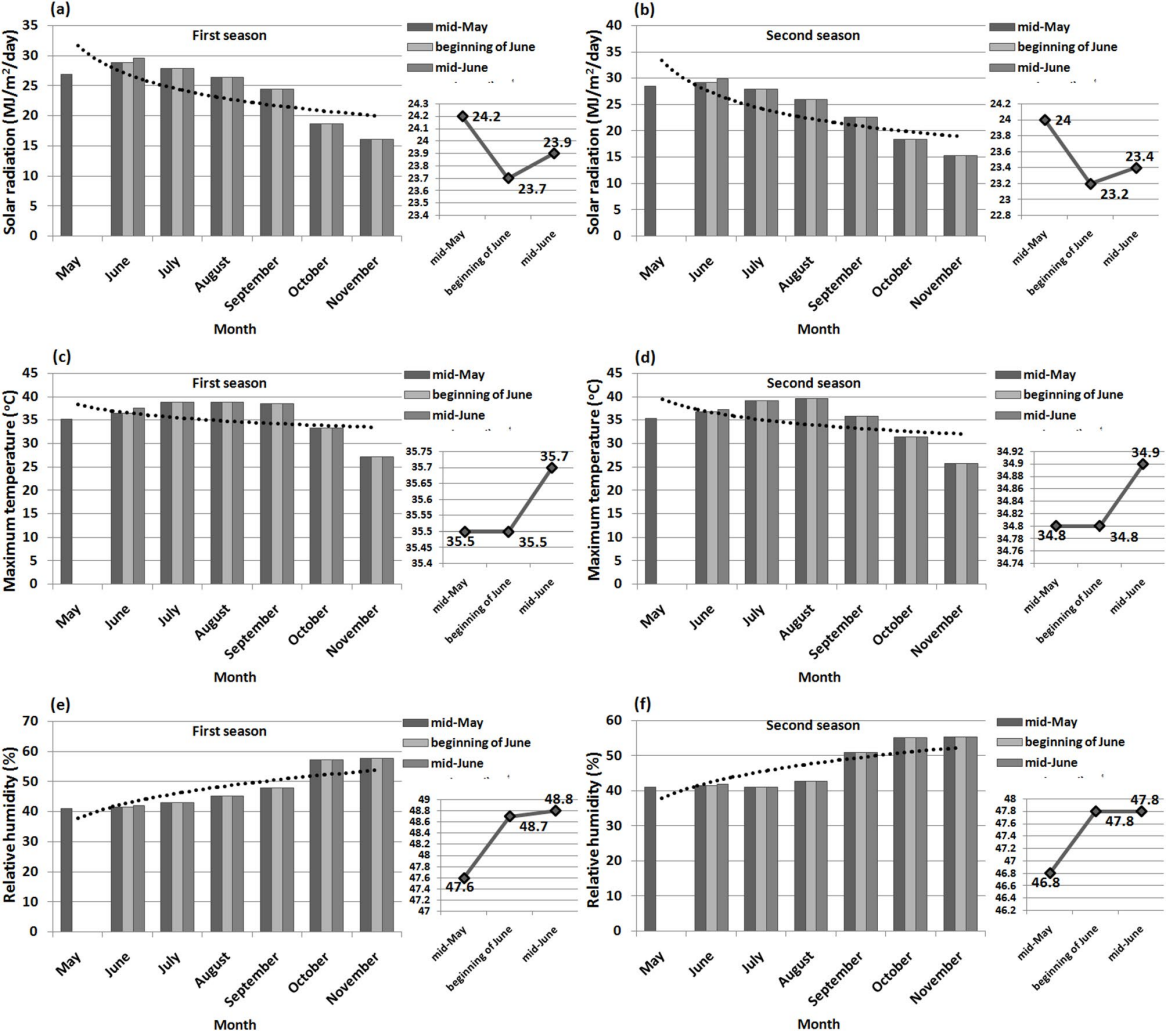
Schematic diagrams showing meteorological data of (**a **and **b**) solar radiation, (**c **and** d**) maximum temperature, and (**e** and **f**) relative humidity in both seasons.

Low levels of organic matter and available nutrients in the soil can decrease resistance to pests, leading to higher infestations of soybean stem flies in soybean fields^12^. Figure 1 shows the solar radiation, maximum temperature, and relative humidity for two summer seasons. The data was obtained from the POWER Docs^13^ website statistics, enabling a thorough analysis of weather conditions.

### Experimental design and agricultural techniques

The preceding winter crop was wheat in both seasons. The experiment utilized a split-plot design with three replications. Planting dates were assigned to the main plots, and soybean varieties were distributed to subplots. Each plot measured 10.8 m^2^ and consisted of six ridges, each 3.0 m long and 0.6 m wide. Randomization was employed to minimize bias and ensure the validity of the results. This design allowed for a comprehensive evaluation of the effects of planting dates and soybean varieties on crop performance. Replication was used to reduce variability within the experiment, enhancing the reliability of the data collected. Additionally, a blocking strategy was implemented to account for potential variations in soil fertility and environmental factors. The structured framework provided by the split-plot design facilitated a thorough and controlled study of the factors influencing crop yield. Soybean varieties were seeded at a density of 20 plants per meter in single rows along ridges. The study assessed the performance of five indeterminate soybean varieties (Table 1), including four Egyptian varieties (Giza 21, Giza 22, Giza 35, Giza 111) and one American variety (Crawford), along with one determinate variety (Dr-101), in response to soybean stem fly infestation across three planting dates (early and late). The aim was to determine if the resistance to soybean stem flies observed in Egyptian determinate varieties during early planting dates (May) persisted during late planting dates (June). The study also included the American variety Crawford to compare its performance with the Egyptian varieties under different planting conditions. Normal recommended cultural practices for growing soybean varieties were used. Plots were uniformly fertilized with all recommended nutrients. Chemical control was entirely avoided.

### Studied traits

Pubescence features of soybean stem were observed on six different varieties with varying levels of insect infestation and pubescence ratings, 75 days after sowing. Pubescence features of soybean stems were observed at 75 days after sowing due to hormonal changes that occur during the plant’s growth cycle. These changes can affect the plant’s nutrient and water absorption, ultimately impacting its growth and yield potential. Three phenotypes of pubescence density were identified: dense, normal, and sparse^14^. Pubescence length (μm), number of pubescences per 500 μm, and pubescence density were measured to assess stem pubescence characteristics. Stem pubescence traits were analyzed using the SEM Model Quanta 250 FEG in the Egyptian Mineral Resources Authority Central Laboratories Sector.

Stem anthocyanins content (mg/100 g FW) of six soybean varieties were analyzed after 75 days. The anthocyanins content (mg/100 g FW) of six soybean varieties was analyzed using the spectrophotometric method through the extraction of pigments with acidified methanol. Variety A had the highest anthocyanins content, while variety F had the lowest. This data is useful for choosing soybean varieties with greater antioxidant properties for consumption or breeding. This analysis was performed by Food Technology Research Department, ARC, Giza, Egypt. Leaf total phenols content (mg/100 g DW) of six soybean varieties was analyzed through a spectrophotometric method. This concentration of sample was calculated by measuring the absorbance at 765 nm and using a standard curve of gallic acid. This analysis was performed by Cairo University Research Park, Faculty of Agriculture, Cairo University, Giza, Egypt.

To assess tunneling in the stem (%) caused by larvae, six plants per plot were randomly taken at maturity (R8). There were steps to assess the tunnel length at maturity (cm): (1) the plant was separated from the roots by cutting off the roots at the base using pruning shears. (2) the plant stem lengthwise was split. (3) the plant stem was visually examined then the tunnel length was measured from the start to the end of the tunnel. The tunnel length (cm) relative to plant height (cm) was measured to determine tunnel damage (%). Observations were made on tunnel damage following the procedure outlined by Upadhyay^15^. Tunnel damage (%) was calculated as follows: Tunnel damage (%) = (Length of tunnel ‘cm’ / Plant height ‘cm’) × 100. In both seasons, the tolerance/susceptibility of different soybean varieties to soybean stem fly infestation was assessed during the first, second, third, and fourth weeks after 45 days from planting. This evaluation was carried out using the plant phenology from vegetative growth reproductive stages at the 7th, 8th, 9th, and 10th weeks after planting, which correspond to 53 days, 60 days, 67 days, and 75 days from planting. Six soybean plants were randomly selected from each plot along the diagonals, and the population density of soybean stem flies in their incomplete stages was evaluated at these intervals. Infected stems often exhibit a red (and sometimes pale) interior, with larvae or pupae found within a distinct zigzag tunnel. Larvae and pupae are most commonly found in the vegetative growth and early reproductive stages of soybean plants^12^. The scoring method for evaluating infestation levels is based on the number of larvae and pupae found within the stems. Higher scores indicate a more severe infestation. This method provides a quantitative assessment of soybean stem fly infestation in various plots and can guide pest management strategies. The infestation percentage was recorded following the methodology outlined by Rajashekar et al.^16^. As we did not use pesticides in these tests, there were no adverse effects on humans, plants, or insects, in line with ethical standards. Our study focused on planting resistant varieties. This approach minimizes environmental harm and aligns with sustainable farming practices.

To study varietal differences across three planting dates and induce protein in soybean stem flies, SDS-PAGE was used to separate total soluble proteins. The total soluble proteins were analyzed using SDS-PAGE to characterize six varieties after sixty days of planting. The 60-day time point was chosen to allow for adequate time for gene expression induction and clear separation of protein patterns. The findings offer valuable insights into the influence of planting date on protein expression in soybean varieties, following the method recommended by UPOV^17^. Variation in protein pattern via the appearance of new bands indicated enhancement of gene expression. This analysis was performed Cairo University Research Park, Faculty of Agriculture, Cairo University, Giza, Egypt. Due to limitations in the imaging equipment available during the experiment, we regret any inconvenience this may have caused. To address this issue, future studies will include full-length blot images to ensure transparency and allow readers to thoroughly assess the data’s quality and validity, thereby enhancing the credibility of the research findings and strengthening the overall impact of the study. As a result of space constraints in the main manuscript, this figure has been moved to a supplementary file. This decision was made to prioritize the clarity and completeness of the data for readers who may need a more detailed examination of the images. After reaching physiological maturity (R8) for each variety in each planting date, ten randomly selected plants were chosen from the central four rows of each plot to record the plant height (cm), number of pods per plant and seed yield per plant (g). The number of days from planting to physiological maturity, defined as the point when 95% of pods have reached their mature color, was determined for each plot by counting the days until all plants in the plot reached physiological maturity. Seed yield (kg) was determined for each plot according to Fehr and Caviness^18^ by harvesting the central four rows at the R8 stage, measuring seed moisture, and adjusting yields to 13% moisture content (130 g/kg). A 100-seed sample was dried at 60 °C until a constant weight was reached, then weighed and adjusted to 13% moisture content.

### Statistical analysis

The study utilized analysis of variance (ANOVA) and mixed models to analyze the effects of planting dates and soybean varieties on the measured variables^19^. ANOVA was performed using Mstat Software (version 8.0.1, URL link: Mstat version 8.0.1 for Windows-14.8 M), while mixed models allowed for random effects. Figures 3 and 4 depict the planting of six soybean varieties in experiments carried out on three different dates, along with the general means. Varieties with the same letters show no significant difference within each date, whereas those with different letters indicate a significant distinction. This facilitates a straightforward comparison of the performance of each variety across the three planting dates. The least significant differences (L.S.D.) test was used for mean comparisons^20^.

## Results

The genetic makeup of soybean varieties determines their characteristics, such as yield potential and maturity, which in turn affects their resistance to pest infestation^9^. Dr-101 (MG-V) and Giza 35 (MG-III) exhibited minimal seed weight loss, indicating high pest resistance. On the other hand, varieties of MG-IV, including Giza 21, Giza 22, and Giza 111, showed moderate resistance^9^. This suggests that specific genetic traits in these varieties contribute to their ability to withstand pest pressures. These findings imply that soybean varieties with these genetic traits may provide better protection against pests, leading to higher yields. In contrast, Crawford (MG-IV) was found to be susceptible^9^, indicating a potential vulnerability to soybean stem fly infestation. Testing various soybean varieties for their resistance to soybean stem fly infestation across three different planting dates can provide valuable insights into identifying the most suitable planting date that maximizes resistance and minimizes crop damage. This information could be beneficial for farmers in optimizing their planting schedules and ultimately increasing soybean yield.

### Pedigree of six soybean varieties and their origins

Table 1 shows the six soybean varieties used in the study, including four Egyptian and two American varieties. Dr-101 exhibits determinate growth, while the remaining varieties show indeterminate growth. These varieties span maturity groups III to V, providing a diverse range for studying soybean growth and development under soybean stem flies infestation in the field. The second planting date experienced the lowest average solar radiation exposure for soybean plants during their growth and development (Fig. 1). Mean maximum and minimum temperatures, as well as relative humidity, increased with the delay in planting date.

### Stem pubescence density

**Fig. 2:**
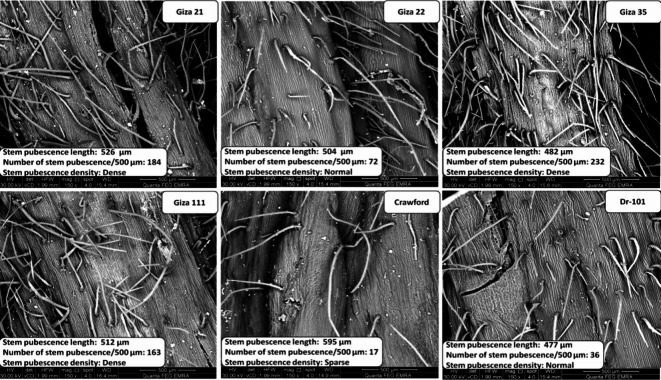
Different types of pubescence density on stem longitudinal section of six soybean varieties at 500 μm. LSD 0.05 Stem pubescence length: 68.08, LSD 0.05 Number of stem pubescence/500 µm: 49.16.

Significant variations were observed in the density and length of pubescences per 500 μm among different soybean varieties (Fig. 2). Crawford’s pubescences were 13.11% longer than those of Giza 21, 18.05% longer than Giza 22, 23.44% longer than Giza 35, 16.21% longer than Giza 111, and 24.73% longer than Dr-101. The highest number of stem pubescences per 500 μm was found in Giza 111, Giza 21, and Giza 35 (163, 184, and 232 µm, respectively). There was a positive correlation between the density of pubescence and the number of pubescences on soybean plants, while a negative correlation was observed between pubescence length and density^21^. Higher pubescence density in Giza 21, Giza 35, and Giza 111 likely contributes to their reduced susceptibility to stem fly infestations. In contrast, Crawford had a lower stem pubescence density, while Giza 22 and Dr-101 exhibited a normal stem pubescence density, as shown in Fig. 2.

### Stem anthocyanins and leaf total phenol contents

**Fig. 3:**
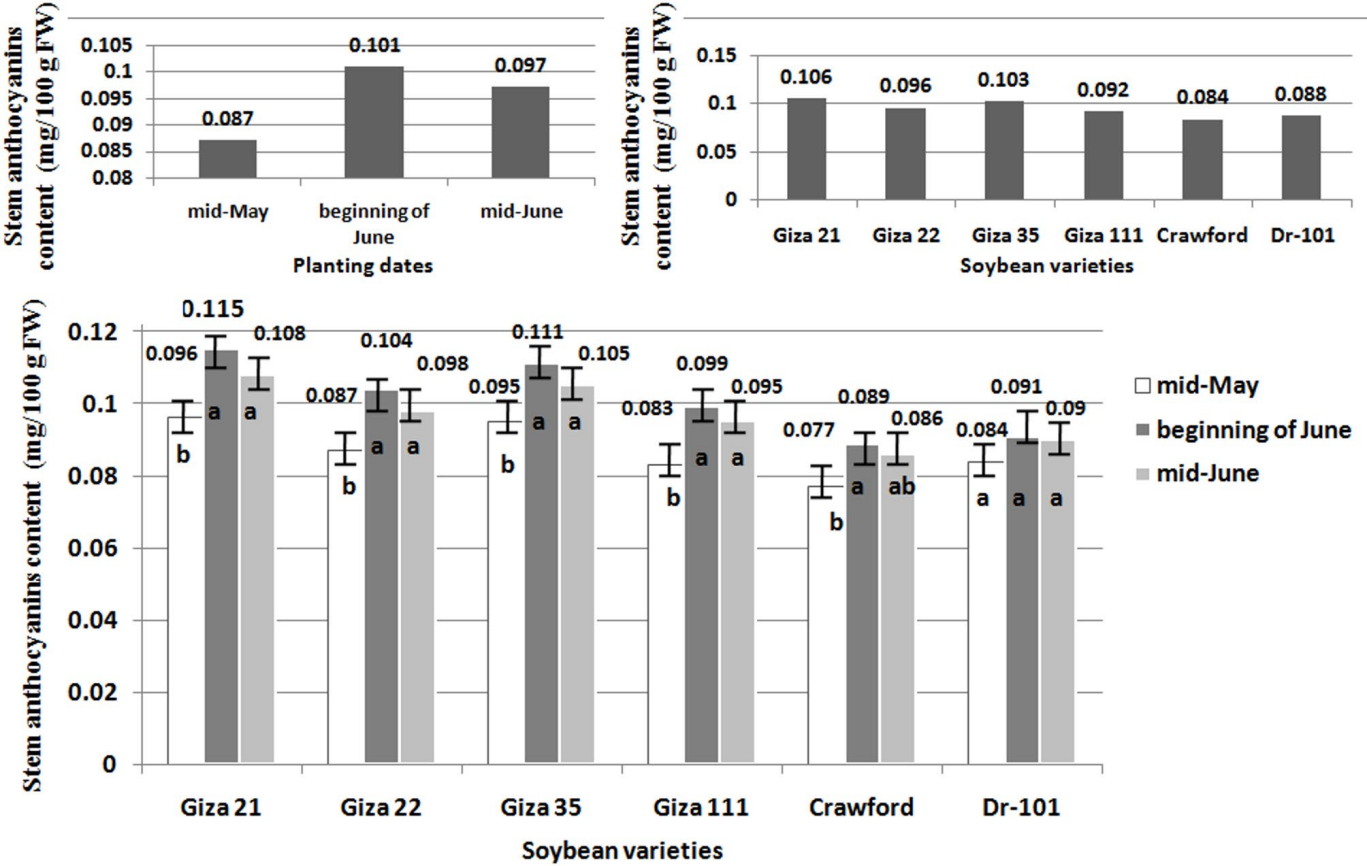
Effect of planting dates, soybean varieties, and their interactions on stem anthocyanins content. LSD 0.05 Planting dates: 0.01, LSD 0.05 Soybean varieties: 0.008, LSD 0.05 Interaction: 0.01.

**Fig. 4:**
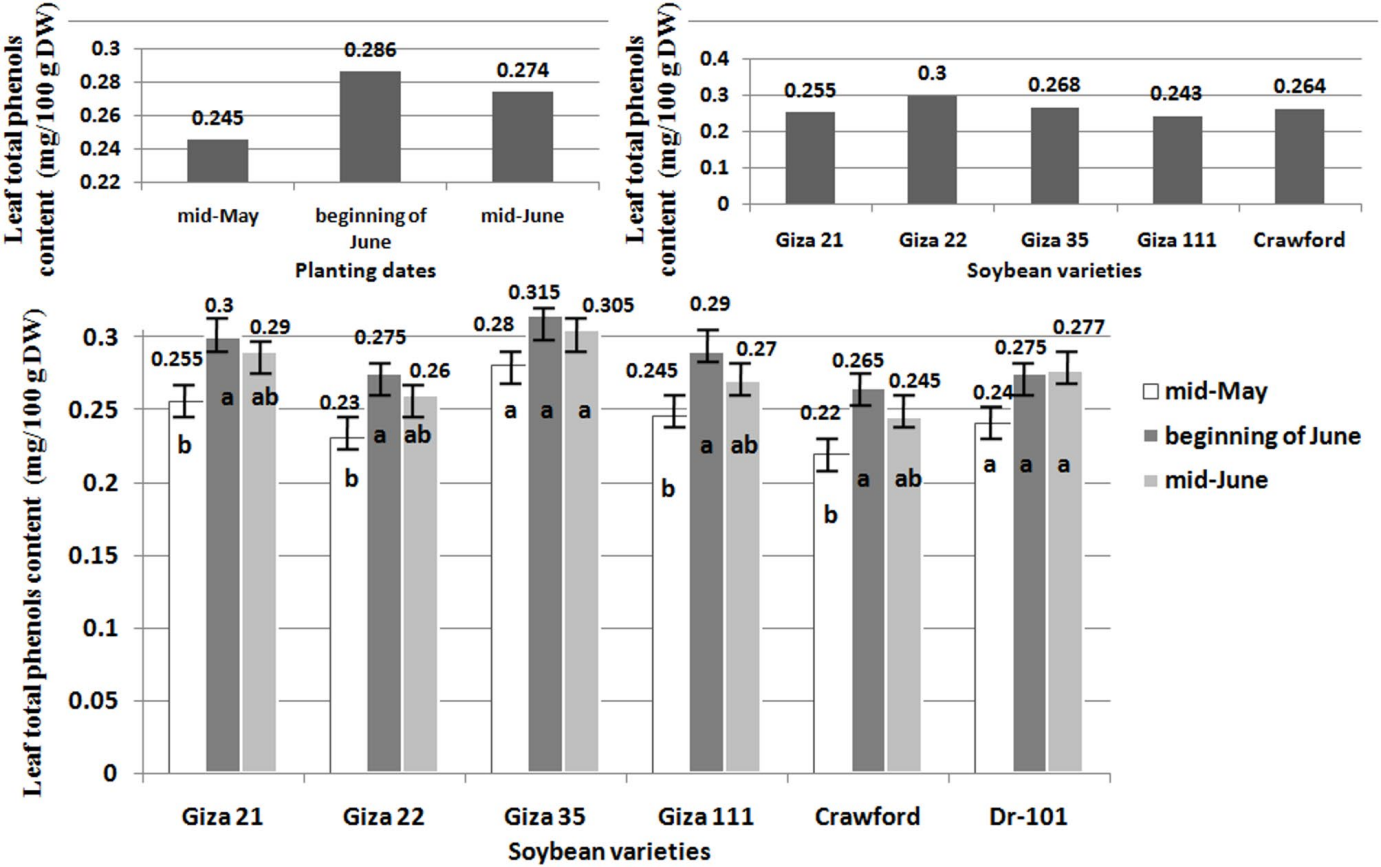
Effect of planting dates, soybean varieties, and their interactions on leaf total phenols content. LSD 0.05 Planting dates: 0.03, LSD 0.05 Soybean varieties: 0.02, LSD 0.05 Interaction: 0.04.

Planting soybeans at the beginning of June resulted in the highest concentrations of anthocyanins, recording 0.101 mg/100 g FW, which was a significant increase of 16.09 compared to planting soybeans at mid-May, which had a lower concentration of stem anthocyanins at 0.087 mg/100 g FW (Figs. 3 and 4). Similarly, planting soybeans at the beginning of June showed the highest concentrations of total phenols, recording 0.286 mg/100 g DW, with a significant increase of 16.73 compared to planting soybeans at mid-May, which had a lower concentration of stem total phenols at 0.245 mg/100 g DW. In contrast, planting soybeans at mid-June resulted in lower concentrations of both anthocyanins and total phenols, with values of 0.097 mg/100 g FW and 0.274 mg/100 g DW, respectively. Giza 21 and Giza 35 showed the highest concentrations of anthocyanins, with 0.106 mg/100 g FW and 0.103 mg/100 g FW, respectively (Figs. 3 and 4). This represented a significant increase of 26.19 and 22.61% compared to Crawford, which had a lower concentration of stem anthocyanins at 0.084 mg/100 g FW. Additionally, Giza 22 had the highest concentrations of total phenols at 0.300 mg/100 g DW, showing a significant increase of 23.45% compared to Giza 111, which had a lower concentration of stem total phenols at 0.243 mg/100 g DW. When planted at the beginning of June instead of mid-May or mid-June, all evaluated soybean varieties showed increased levels of anthocyanins and total phenols (Figs. 3 and 4).

The planting date did not affect the total phenol content in Giza 35. For Dr-101, the planting date did not impact the anthocyanins and total phenol contents. However, planting Giza 21, Giza 22, Giza 111, and Crawford at the beginning of June resulted in increased anthocyanins and total phenol content compared to planting in mid-May. There were no significant differences in anthocyanins and total phenol contents when planting in mid-June.

### Tunnel damage

**Fig. 5:**
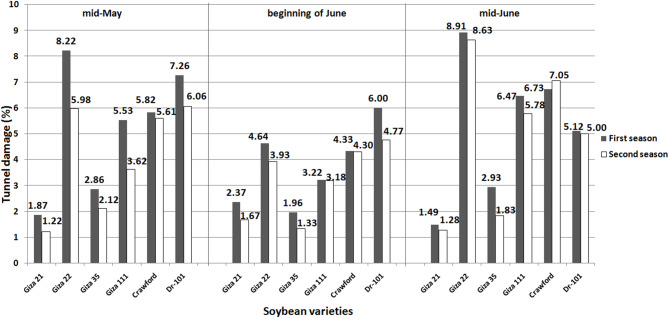
Tunnel damage in soybean varieties due to infestation by soybean stem flies in fields planted on three different dates. LSD 0.05 Planting dates: ns in both seasons, LSD 0.05 Soybean varieties: 2.49 in the first season and 2.03 in the second season, LSD 0.05 Interaction: 3.68 in the first season and 3.31 in the second season.

While planting dates did not significantly affect average tunnel damage, Giza 22 and Crawford exhibited increased damage when planted in mid-May or mid-June (Fig. 5). Tunnel damage levels for Giza 22 significantly decreased when planted at the beginning of June, with reductions of 43.55 and 47.92% in the first season compared to mid-May and mid-June, and 34.28% and 54.46% in the second season compared to mid-May and mid-June, respectively. Crawford showed decreased tunnel damage of 25.60 and 35.66% in the first season, and 23.35 and 39.00% in the second season when planted at the beginning of June instead of mid-May or mid-June.

Giza 21 experienced a decrease in tunnel damage levels of 21.09 and 37.13% in the first season and 26.94 and 23.35% in the second season when planted in mid-May and mid-June, respectively. Similarly, Giza 35 showed lower tunnel damage levels of 31.46 and 33.10% in the first season, and 37.26 and 27.32% in the second season when planted at the beginning of June compared to mid-May and mid-June, respectively. Giza 111 also exhibited significant reductions in tunnel damage of 41.77% and 50.23% in the first season and 12.15 and 44.98% in the second season when planted at the beginning of June compared to mid-May and mid-June, respectively. Dr-101 planted at the beginning of June reduced tunnel damage by 17.35% in the first season and 21.28% in the second season compared to mid-May planting. Additionally, soybeans planted at the beginning of June in the second season had a 4.60% decrease in tunnel damage compared to mid-June planting. However, Dr-101 planted at the beginning of June showed a 17.18% increase in tunnel damage in the first season compared to mid-June planting.

### Soybean stem fly assemblages

**Fig. 6:**
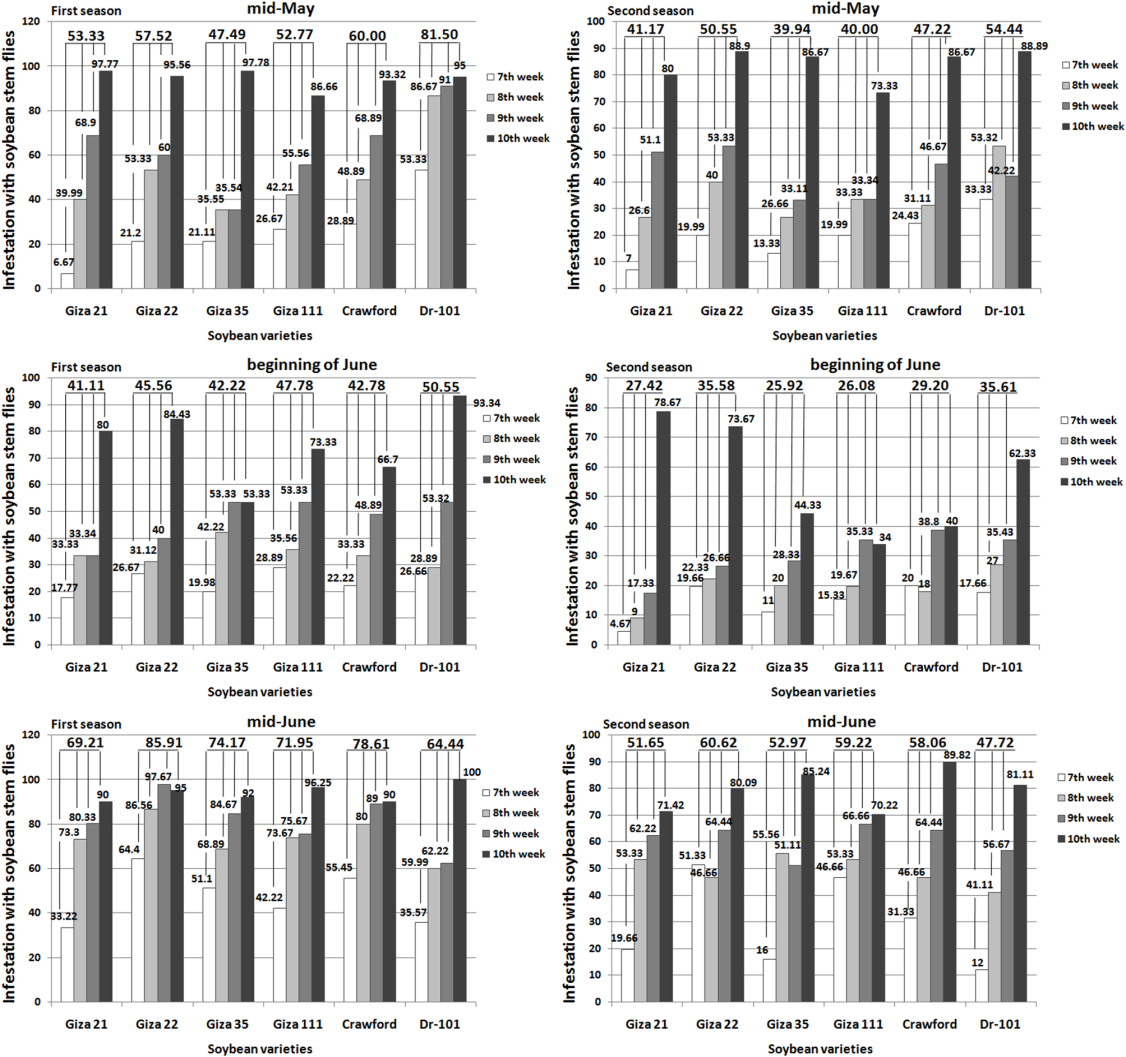
Weekly variations in infestation levels of soybean stem flies on different soybean varieties planted on three different dates. First season, mid-May: LSD 0.05 mean: 13.3, LSD 0.05 7th week: 16.3, LSD 0.05 8th week: ns, LSD 0.05 9th week: 15.4, and LSD 0.05 10th week: ns. Second season, mid-May: LSD 0.05 mean: 9.1, LSD 0.05 7th week: 12.2, LSD 0.05 8th week: 17.0, LSD 0.05 9th week: 12.5, and LSD 0.05 10th week: 8.2. First season, beginning of June: LSD 0.05 mean: ns, LSD 0.05 7th week: ns, LSD 0.05 8th week: ns, LSD 0.05 9th week: 14.3, and LSD 0.05 10th week: 22.8. Second season, beginning of June: LSD 0.05 mean: ns, LSD 0.05 7th week: ns, LSD 0.05 8th week: ns, LSD 0.05 9th week: 13.1, and LSD 0.05 10th week: 21.7. First season, mid-June: LSD 0.05 mean: 8.4, LSD 0.05 7th week: ns, LSD 0.05 8th week: ns, LSD 0.05 9th week: ns, and LSD 0.05 10th week: ns. Second season, mid-June: LSD 0.05 mean: 8.6, LSD 0.05 7th week: ns, LSD 0.05 8th week: ns, LSD 0.05 9th week: ns, and LSD 0.05 10th week: ns.

The assemblages of soybean stem flies increased from the 7th to the 10th week for each planting date in both seasons (Fig. 6). The trends were consistent across different planting dates and soybean varieties, with varying degrees of increase in stem fly assemblages over time. When soybeans were planted in mid-May for both seasons, significant differences were observed in the assemblages of soybean stem flies among varieties at weeks 7 and 9. In the second season, when soybeans were planted in mid-May, distinct differences in the assemblages of soybean stem flies were observed between cultivars at weeks 8 and 10. When soybeans were planted at the beginning of June for both seasons, significant differences were observed in the assemblages of soybean stem flies among varieties at weeks 9 and 10. When soybeans were planted in mid-June for both seasons, no significant differences were observed in the assemblages of soybean stem flies among varieties at weeks 7, 8, 9, and 10.

The abundance of soybean stem fly was lower on soybean varieties Giza 21, Giza 35, Giza 111, and Crawford when planted in mid-May compared to mid-June (Fig. 6). When Giza 21 was planted in mid-June instead of mid-May, the abundance increased by 29.77% in the first season and 25.45% in the second season. Similarly, planting Giza 35 in mid-June resulted in a 56.18% increase in the first season and a 32.62% increase in the second season. Planting Giza 111 in mid-June led to a 36.34% increase in the first season and a 48.05% increase in the second season. Planting Crawford in mid-June led to a 31.01% increase in the first season and a 22.95% increase in the second season. In contrast, planting Dr-101 in mid-June resulted in a 20.93% decrease in the first season and a 12.34% decrease in the second season.

### SDS—protein analysis

A total of 14 bands with molecular weights ranging from 20 to 400 kDa were identified (Table 2). Among these bands, 7 were polymorphic (50.0%) and 7 were monomorphic (50.0%). The total number of protein bands ranged from 27 to 21. Giza 21, Giza 22, Giza 35, Dr-101, Giza 111, and Crawford had 27, 26, 24, 21, and 21 bands, respectively. Planting Giza 21 in mid-May resulted in the appearance of a distinctive 240 kDa band.

**Table 2:** Densitometric analysis of SDS-protein PAGE for leaf of six soybean varieties through three planting dates infested by soybean stem flies.

MW (KD_a_)	Mid-May						Beginning of June						Mid-June					
	1	2	3	4	5	6	1	2	3	4	5	6	1	2	3	4	5	6
400	–	–	–	–	–	–	–	–	–	–	–	–	–	–	–	–	–	–
350	+1	+1	+1	–	–	–	+1	+1	+1	–	+1	+1	+1	+1	–	–	–	+1
300	+ 1	–	–	–	–	–	+1	–	–	–	–	–	+1	–	–	–	–	–
240	+1	+2	+3	+4	+3	+3	–	+4	+4	+4	+4	+4	–	–	+4	–	+3	+3
210	–	+2	–	–	–	–	–	+3	–	–	–	–	–	–	–	–	–	–
180	+4	+4	+4	+3	+3	+3	+4	+3	+3	+3	+3	+3	+4	+3	+ 3	+3	+3	+3
150	+2	+2	+2	+2	+2	+2	+2	–	–	–	–	–	–	–	–	–	–	–
100	+4	+4	+4	+4	+4	+4	+4	+4	+4	+4	+4	+4	+4	+4	+4	+4	+4	+4
90	+2	+2	+2	+2	–	–	+2	+2	+2	+2	+2	+2	+2	+2	+2	+2	–	+2
75	+4	+4	+4	+4	+4	+4	+ 4	+4	+4	+4	+4	+4	+4	+4	+4	+4	+4	+4
65	+4	+4	+4	+4	+4	+4	+4	+4	+4	+4	+4	+4	+4	+4	+4	+4	+4	+4
55	–	–	–	–	–	–	–	–	–	–	–	–	–	–	–	–	–	+4
40	+4	+4	+4	+4	+4	+4	+4	+4	+4	+4	+4	+4	+4	+4	+4	+4	+4	+4
20	–	–	–	–	–	–	–	–	–	–	–	–	–	–	–	–	–	–
Total	10	10	9	8	7	7	9	9	8	7	8	8	8	7	7	6	6	9

+ 4: v.v. dark, + 3: dark, + 2: Clear, + 1: Faint, –: absent, MW = Molecular weight, M = Marker, 1 = Giza 21, 2 = Giza 22, 3 = Giza 35, 4 = Giza 111, 5 = Crawford, 6 = Dr-101. The densitometric analysis of SDS-protein PAGE for the leaf sample showed a very dark band at position + 4, indicating a high concentration of the protein of interest. This suggests that the leaf sample has a significant amount of the target protein compared to other bands on the gel. In contrast, there was an absence of any band at position -, suggesting that this protein was not present in the sample.

Planting Giza 21 or Giza 22 in mid-May and the beginning of June led to the appearance of a distinctive 150 kDa band. Giza 22 showed a distinctive 210 kDa band when planted in mid-May. Giza 35, Giza 111, or Crawford exhibited a distinct 150 kDa band when planted in mid-May. Giza 111 displayed a distinctive 240 kDa band when planted in mid-May and the beginning of June, but Crawford had a distinct 90 kDa band when planted at the beginning of June. Dr-101 showed a distinct 350 kDa band when planted at the beginning of June and mid-June, a 150 kDa band when planted in mid-May, and a 55 kDa band when planted in mid-June.

### Seed yield and yield components

Planting soybeans in mid-May resulted in an increase in days to maturity by 2.04 and 2.87%, plant height by 2.97 and 3.38%, number of pods per plant by 9.35 and 10.11%, seed yield per plant by 15.00 and 17.50%, 100-seed weight by 16.50 and 10.58%, and seed yield per plot by 7.17 and 9.85% in the first and second seasons, respectively, compared to planting soybeans at the beginning of June (Table 3). Additionally, planting soybeans in mid-May resulted in an increase in days to maturity by 6.29 and 8.04%, plant height by 8.46 and 8.56%, number of pods per plant by 16.91 and 15.59%, seed yield per plant by 19.75 and 19.32%, 100-seed weight by 22.27 and 20.09%, and seed yield per plot by 12.88 and 10.94% in the first and second seasons, respectively, compared to planting soybeans in mid-June. Giza 35 was the earliest mature variety in both seasons, followed by Crawford, while Dr-101 was the late-maturing variety. Giza 35 reached maturity in 104 days in the first season and 90.33 days in the second season, showing a significant difference in maturity time compared to Dr-101. Dr-101 took 161.66 days to mature in the first season and 153.33 days in the second season. Giza 111 was the shortest variety, measuring 62.40 cm in the first season and 79.69 cm in the second season, with Giza 22 being the second shortest. The tallest varieties were Giza 21 and Giza 35, reaching heights of 104.37 cm and 103.02 cm in the first season, respectively, and 119.77 cm and 113.08 cm in the second season, respectively Although pod production per plant was highest for Giza 22 in both seasons, but Giza 111 and Dr-101 had the highest seed yields per plant in the first season at 32.94 and 31.91 g, respectively, and in the second season at 29.11 and 27.40 g, respectively. In the first and second seasons, Giza 111 had the highest 100-seed weight, measuring 20.47 g and 17.52 g, respectively, followed by Giza 35 with 17.07 g in the first season and 14.58 g in the second season. Giza 111 and Giza 35 also had the highest seed yield per plot, with Giza 111 producing 2.62 kg and Giza 35 producing 2.60 kg in the first season, and 2.26 kg and 2.33 kg in the second season, respectively. Mid-June planting resulted in the shortest maturity time for Giza 35 and Dr-101, while Giza 22 and Giza 35 showed increased pod production (Table 3).

**Table 3:** Effect of planting dates, soybean varieties, and their interactions on seed yield and yield components. Giza 35 (MG-III), Giza 21 (MG-IV), Giza 22 (MG-IV), Giza 111 (MG-IV), Crawford (MG-IV), and Dr-101 (MG-V).

Planting date	Soybean variety	Days to maturity (no.)	Plant height (cm)	Pods/plant (no.)	Seed yield/plant (g)	100-seed weight (g)	Seed yield/plot (kg)
First season							
Mid-May	Giza 21	134.00	106.82	66.23	29.43	16.28	2.44
	Giza 22	134.00	79.02	96.58	34.43	15.35	2.67
	Giza 35	108.00	104.76	90.67	32.91	18.90	2.75
	Giza111	136.00	63.30	77.85	36.26	22.13	2.78
	Crawford	135.00	94.51	67.45	32.47	19.32	2.18
	Dr-101	163.00	96.39	62.74	32.77	17.72	2.42
Mean		135.00	90.80	76.92	33.04	18.28	2.54
Beginning of June	Giza 21	132.00	105.45	61.56	25.92	15.19	2.37
	Giza 22	131.00	75.42	94.98	29.79	10.69	2.54
	Giza 35	105.00	102.08	78.71	30.72	17.61	2.64
	Giza111	134.00	62.06	74.49	33.03	20.46	2.72
	Crawford	129.00	92.37	57.93	24.50	15.33	1.98
	Dr-101	163.00	91.73	54.42	28.42	14.91	2.00
Mean		132.30	88.18	70.34	28.73	15.69	2.37
Mid-June	Giza 21	128.00	100.86	54.34	22.97	12.79	2.18
	Giza 22	127.00	72.93	86.67	25.20	11.82	2.37
	Giza 35	99.00	102.23	73.21	26.35	14.70	2.42
	Giza111	128.00	61.84	69.45	29.55	18.84	2.35
	Crawford	121.00	66.83	50.60	26.96	12.68	1.72
	Dr-101	159.00	97.59	60.51	34.54	18.88	2.45
Mean		127.00	83.71	65.79	27.59	14.95	2.25
Average of soybean variety	Giza 21	131.33	104.37	60.71	26.10	14.75	2.33
	Giza 22	130.66	75.79	92.74	29.80	12.62	2.52
	Giza 35	104.00	103.02	80.86	29.99	17.07	2.60
	Giza111	132.66	62.40	73.93	32.94	20.47	2.62
	Crawford	128.33	84.57	58.66	27.97	15.77	1.96
	Dr-101	161.66	95.23	59.22	31.91	17.17	2.28
LSD 0.05 planting dates		4.19	2.80	5.08	1.23	1.40	0.06
LSD 0.05 soybean varieties		3.15	3.20	4.04	1.08	0.91	0.05
LSD 0.05 interaction		5.96	ns	6.84	1.67	1.56	0.27

Second season							
Mid-May	Giza 21	125.00	123.03	58.68	25.05	14.49	2.18
	Giza 22	123.00	91.99	89.56	30.21	12.91	2.36
	Giza 35	96.00	117.51	82.84	28.68	16.26	2.43
	Giza111	126.00	82.68	71.75	32.34	18.87	2.40
	Crawford	126.00	106.82	58.41	27.10	16.49	1.88
	Dr-101	156.00	107.11	56.73	27.80	15.67	2.13
Mean		125.33	104.85	69.66	28.53	15.78	2.23
Beginning of June	Giza 21	123.00	119.18	53.05	21.52	13.53	2.09
	Giza 22	121.00	88.90	88.21	25.79	10.20	2.21
	Giza 35	91.00	112.32	72.28	26.17	15.18	2.33
	Giza111	123.00	78.54	66.85	29.23	17.75	2.24
	Crawford	119.00	104.85	52.15	19.10	13.89	1.64
	Dr-101	154.00	104.77	47.03	23.90	15.12	1.68
Mean		121.83	101.42	63.26	24.28	14.27	2.03
Mid-June	Giza 21	119.00	117.10	48.10	18.87	10.60	1.99
	Giza 22	115.00	86.92	81.03	21.47	10.79	2.04
	Giza 35	84.00	109.42	68.69	23.47	12.30	2.23
	Giza111	117.00	77.87	63.19	25.78	15.95	2.13
	Crawford	111.00	78.01	45.12	23.38	11.22	1.38
	Dr-101	150.00	110.18	55.44	30.51	18.02	2.31
Mean		116.00	96.58	60.26	23.91	13.14	2.01
Average of soybean variety	Giza 21	122.33	119.77	53.27	21.81	12.80	2.08
	Giza 22	119.66	89.27	86.26	25.82	11.30	2.20
	Giza 35	90.33	113.08	74.60	26.10	14.58	2.33
	Giza111	122.00	79.69	67.26	29.11	17.52	2.26
	Crawford	118.66	96.56	51.89	23.19	13.86	1.64
	Dr-101	153.33	107.35	53.06	27.40	16.27	2.04
LSD 0.05 planting dates		3.40	1.31	4.89	1.18	0.71	0.15
LSD 0.05 soybean varieties		2.30	2.42	3.68	0.97	0.58	0.10
LSD 0.05 interaction		5.12	ns	6.46	1.52	1.23	0.24

All tested soybean varieties exhibited a decrease in seed yields and 100-seed weight when planted later in mid-June, except for Dr-101, which showed a decrease in seed yields and 100-seed weight when planted earlier in mid-May. In terms of seed yield per plant, Giza 111 outperformed Giza 22 when planted in mid-May, with a yield increase of 5.31 and 7.05% in the first and second seasons, respectively. Dr-101 had higher seed yields per plant when planted in mid-June, with yields of 34.54 and 30.51 g in the first and second seasons, respectively, compared to mid-May planting with yields of 32.77 and 27.80 g in the first and second seasons. Regarding 100-seed weight, Giza 111 showed an increase of 14.54 and 14.43% in the first and second seasons, respectively, compared to the second-ranked variety, Crawford. Dr-101 had a higher 100-seed weight when planted in mid-June, with weights of 18.88 and 18.02 g in the first and second seasons, respectively, compared to mid-May planting with weights of 17.72 and 15.67 g in the first and second seasons, respectively. Giza 111 and Giza 22 had higher seed yield per plot compared to Crawford. Giza 111 yielded 2.78 kg in the first season, which was 27.52% higher than Crawford when planted in mid-May. Similarly, Giza 22 yielded 2.75 kg in the first season, which was 26.14% higher than Crawford when planted in mid-May. In the second season, Giza 111 and Giza 22 yielded 2.40 and 2.43 kg, respectively, which were 27.65 and 29.25% higher than Crawford when planted in mid-May. Dr-101 yielded 2.45 kg per plot in the first season and 2.31 kg per plot in the second season, compared to 2.42 kg and 2.13 kg per plot for Dr-101 planted in mid-May. There was no significant difference in seed yield per plot between the two planting dates.

## Discussion

Short and dense pubescence in stem of Giza 21, Giza 35, and Giza 111 supports our hypothesis for resistance pest infestation (Fig. 2). Soybean plants with short and dense pubescence exhibited the highest resistance to common cutworm in soybeans^21^. Camp soybean variety has low trichome density, which decreases attraction and hinders nymph development of *Megacopta cribraria*. Trichomes play a crucial role in reducing insect damage by minimizing contact with the plant’s surface^22–24^. Therefore, variations in stem pubescence density have a significant impact on soybean resistance to stem flies.

Planting soybeans in mid-June may help reduce soybean stem fly populations on certain varieties like Giza 35 and Giza 111. However, planting soybeans of MG-IV and MG-V in mid-May did not increase anthocyanins and total phenols content as expected. Varieties such as Giza 21, Giza 22, Giza 111, and Crawford were negatively impacted by high solar radiation during the growing season (Fig. 1), leading to a decrease in anthocyanins and total phenols synthesis^25,26^, as well as an increase in tunnel damage and pest infestation (Figs. 5 and 6). Crawford, Giza 111, and Giza 22 are known for their chlorophyll content^9,27,28^, with Giza 111 having broad leaves^9,28^ and Giza 22 having narrow leaves^9,27^, aiding in photosynthesis. High temperatures can also reduce the production of these compounds in late-planted MG-IV varieties^29,30^. Dr-101, with a longer maturity period (Table 3), was less impacted by solar radiation (Figs. 3 and 4). Early planting did not reduce tunnel damage and pest infestation in these varieties (Figs. 5 and 6). Planting soybeans later (mid-June) may be a more influential factor in managing soybean stem fly populations than the choice of variety. Dr-101 showed higher levels of anthocyanins and total phenols when planted late, likely due to increased accumulation time (Figs. 3 and 4) as temperatures decrease (Fig. 1). Soybeans from MG-III (Giza 35) supported the hypothesis that early planting reduces tunnel damage and pest infestation (Figs. 5 and 6), with solar radiation not significantly affecting them. Giza 35 matures quickly, countering the negative impact of high temperatures on anthocyanins and phenols. Previous studies have shown that out of 21 soybean genotypes tested for field resistance against stem flies, DSb 34, DLSb 1, KDS 1096, and NRC 197 exhibited significantly lower levels of stem fly infestation and stem tunneling compared to other genotypes, indicating their resistance^31^. Previous research provided a thorough overview of the life cycle, infestation patterns, and various management strategies, including planting date, for the soybean stem fly^32^.

High temperatures can reduce the production of these compounds in soybeans, especially in late-planted MG-III varieties. Soybean stem fly populations may increase during specific times, potentially affecting soybean growth and yield. Understanding environmental conditions and plant growth stages can help determine planting dates and minimize pest damage. Implementing integrated pest management strategies can maximize crop productivity. The findings are linked to known factors that influence stem fly dynamics, such as temperature and host plant phenology. High solar radiation, temperature, and relative humidity in late planting dates positively affected soybean stem flies due to their preference for warm and humid conditions^33^. The abundance of host plants during late planting dates may have also contributed to the increased presence of soybean stem flies. Conversely, low solar radiation, temperature, and relative humidity in early planting dates negatively affected soybean stem flies as they prefer cooler and drier conditions^33^. This highlights the importance of planting date and environmental factors in determining the population dynamics of soybean stem flies in agricultural fields. Farmers in Egyptian soybean fields are concerned about the limited chemical control options available for managing soybean stem flies due to restrictions on certain pesticides. As a result, research is exploring alternative strategies, such as adjusting planting dates, to effectively control the pest population. These findings can inform regional pest management strategies by providing evidence-based recommendations for optimizing soybean production and minimizing economic losses associated with soybean stem fly infestations. Additionally, collaboration between researchers, farmers, and agricultural extension services can facilitate the adoption of integrated pest management practices to ensure sustainable soybean cultivation in Egypt.

Breeding soybean varieties with enhanced anthocyanins and phenolic profiles to boost resistance against soybean stem flies is a promising research avenue that could revolutionize pest control methods for soybean cultivation. By strengthening soybeans’ innate defenses through selective breeding, farmers could potentially decrease reliance on chemical insecticides, thereby enhancing crop health and productivity.

Proteins play a crucial role in protecting soybean plants from stem fly infestation. Different soybean varieties exhibit genetic diversity, as shown in distinct protein band patterns (Table 2). This diversity indicates variations in gene expression or protein composition. Each soybean variety has its unique protein band patterns. The expression of soybean protein in different soybean varieties is influenced by planting dates, revealing distinct patterns.

The results in Table 3 indicate that planting soybeans in mid-May allows for a longer growing period and better climatic conditions (Fig. 1), resulting in higher yields and improved plant growth. Dr-101 has a longer maturity period compared to other varieties tested. Early planting can delay maturity due to extended post-flowering photoperiods^34^. Meteorological conditions at planting can affect maturity rates^35^. Delayed maturity can increase dry matter accumulation and seed weight during seed-filling. Shorter vegetative and reproductive stages may reduce yield by decreasing pod and seed numbers per unit area^36^. Giza 22 excels in pod production compared to Crawford, Giza 111, Dr-101, and Giza 21^19,28^. The tunnel damage in the stem of Giza 111 was minimal (Fig. 5), resulting in enhanced photosynthate transfer and increased seed production. Giza 35 shows rapid dry matter accumulation and resistance to insects, which contributes to its high performance. The lower seed yield of Crawford is attributed to tunnel damage that weakens plant defenses (Figs. 5 and 6). Early planting of MG-III variety (Giza 35) leads to quicker maturity and more dry matter accumulation^27^. MG-V variety (Dr-101) was planted later, there was no decrease in dry matter accumulation compared to earlier planting. This can be attributed to consistent levels of total phenols and leaf anthocyanins (Figs. 3 and 4), which positively impacted tunnel damage and insect resistance (Figs. 5 and 6). Furthermore, factors such as reduced sun radiation, maximum temperature, and consistent humidity in October and November (Fig. 1) also played a role. High temperatures during soybean seed-filling can negatively affect sink size and the ratio of source ability to sink size, resulting in reduced yields. This issue can be addressed by planting late-maturing genotypes at a later time^37^. Giza 111 and Giza 35 exhibit higher seed yield and 100-seed weight when planted early. In general, seed yield is maximized with early planting of MG-III and IV varieties, decreasing with delayed planting^35^.

## Conclusion

Giza 35 and Giza 111 are recommended for boosting productivity and reducing the need for chemical insecticides to control the soybean stem fly in mid-May. In case Egyptian farmers need to postpone soybean planting until mid-June, Dr-101 is a suitable option to tackle the soybean stem fly infestation. By promoting the use of Giza 35 and Giza 111 for early planting and Dr-101 for delayed planting, farmers can adopt sustainable agricultural practices. Policy makers in Egypt should support these suggestions to improve soybean production and reduce environmental impact in the country.

## Supplementary Information

Below is the link to the electronic supplementary material.

Supplementary Material 1

Supplementary Material 2

## Data Availability

The data that support the findings of this study are available from the corresponding author upon reasonable request.

## Abbreviations

MG: Maturity group
SDS-PAGE: Sodium dodecyl-sulfate polyacrylamide gel electrophoresis
kDa: A non-SI unit of mass used to express protein mass. It is equal to 1000 daltons
MJ: Mega joule
FW: Fresh weight
DW: Dry weight
