# Supplementary material for: Impact of planting dates on yield and resistance of soybean varieties to soybean stem fly (Melanagromyza sojae) in Egypt
Source: Sci Rep. 2025 Sep 18;15:32599. doi: 10.1038/s41598-025-19034-2 (PMC12446469; doi:10.1038/s41598-025-19034-2)
Supplement: Supplementary file 1 — Supplementary Material 1 [file 41598_2025_19034_MOESM1_ESM.docx]

**Cover letter for publishing a research article in Scientific Reports**

**Impact of Planting Dates on Yield and Resistance of Soybean Varieties to Soybean Stem Fly (*Melanagromyza sojae*) in Egypt**

## Eman I. Abdel-Wahab^1*^, Magda H. Naroz^2^, Soheir F. Abd El-Rahman^3^

^1^ Food Legumes Research Department, Field Crops Research Institute, Agricultural Research Center, Giza postal box 12619, Egypt; e-mail: [eman00eman70@yahoo.com](mailto:eman00eman70@yahoo.com); ORCID: 0000-0002-2277-7238.

^2^ Economic Entomology and Pesticides Department, Faculty of Agriculture, Cairo University, Giza, Egypt, Giza postal box 12622, Egypt;

^3^ Plant Protection Research Institute, Agricultural Research Center, Dokki, Giza, Egypt

----------------------------------------------------------------------------------------------------------

We are writing for publishing a research paper in Scientific Reports under title: Impact of Planting Dates on Yield and Resistance of Soybean Varieties to Soybean Stem Fly (*Melanagromyza sojae*) in Egypt, this study aims to identify the optimal planting date for soybeans and determine the most productive variety while minimizing infestation by soybean stem flies. **We resubmitted the paper on the manuscript system of Scientific Reports after the editorial board member agreed to consider our appeal and assess our manuscript.**

**Note: all authors are from low income country (Egypt).**

**Thank you for your time and consideration**

**-------------------------------------------------------------------------------------------**

Sincerely yours,

**Eman Ibrahim Abdel-Wahab**

Food Legumes Res. Dept.

Field Crops Res. Inst.

Agric. Res. Center

Giza, Egypt

**Magda Hanna Naroz**

Economic Entomology and Pesticides Dept.

Fac. Agric.

Cairo Univ.

Giza, Egypt

**Soheir F. Abd El-Rahman**

Plant Protection Res. Inst.

Agric. Res. Center

Giza, Egypt
