# Supplementary material for: Impact of planting dates on yield and resistance of soybean varieties to soybean stem fly (Melanagromyza sojae) in Egypt
Source: Sci Rep. 2025 Sep 18;15:32599. doi: 10.1038/s41598-025-19034-2 (PMC12446469; doi:10.1038/s41598-025-19034-2)
Supplement: Supplementary file 2 — Supplementary Material 2 [file 41598_2025_19034_MOESM2_ESM.pdf]

Month	Solar radiation (MJ/m^2/day)			Maximum temperature (˚C)			Relative humidity (%)		
	mid-May	beginning of June	mid-June	mid-May	beginning of June	mid-June	mid-May	beginning of June	mid-June
	First season								
May	26.9	--	--	35.2	--	--	40.9	--	--
June	28.9	28.9	29.6	36.5	36.5	37.6	41.5	41.5	42.0
July	27.9	27.9	27.9	38.8	38.8	38.8	42.9	42.9	42.9
August	26.4	26.4	26.4	38.8	38.8	38.8	45.3	45.3	45.3
Sep.	24.5	24.5	24.5	38.5	38.5	38.5	47.8	47.8	47.8
Oct.	18.7	18.7	18.7	33.3	33.3	33.3	57.2	57.2	57.2
Nov.	16.1	16.1	16.1	27.2	27.2	27.2	57.6	57.6	57.6
Mean	24.2	23.7	23.9	35.5	35.5	35.7	47.6	48.7	48.8
	Second season								
May	28.5	--	--	35.3	--	--	41.0	--	--
June	29.2	29.2	29.9	36.8	36.8	37.2	41.4	41.4	41.8
July	28.0	28.0	28.0	39.2	39.2	39.2	41.1	41.1	41.1
August	25.9	25.9	25.9	39.7	39.7	39.7	42.8	42.8	42.8
Sep.	22.6	22.6	22.6	35.9	35.9	35.9	50.9	50.9	50.9
Oct.	18.4	18.4	18.4	31.5	31.5	31.5	55.1	55.1	55.1
Nov.	15.3	15.3	15.3	25.8	25.8	25.8	55.3	55.3	55.3
Mean	24.0	23.2	23.4	34.8	34.8	34.9	46.8	47.8	47.8

Table S1. Meteorological data of solar radiation, maximum temperature, and relative humidity. This data is essential for understanding weather patterns and evaluating potential soybean varieties for their resistance to soybean stem flies. These factors play a crucial role in plant growth and susceptibility to insects across three planting dates. Analyzing this data allows researchers to identify the most suitable soybean varieties for specific planting dates, leading to improved crop yield and decreased insect infestation.

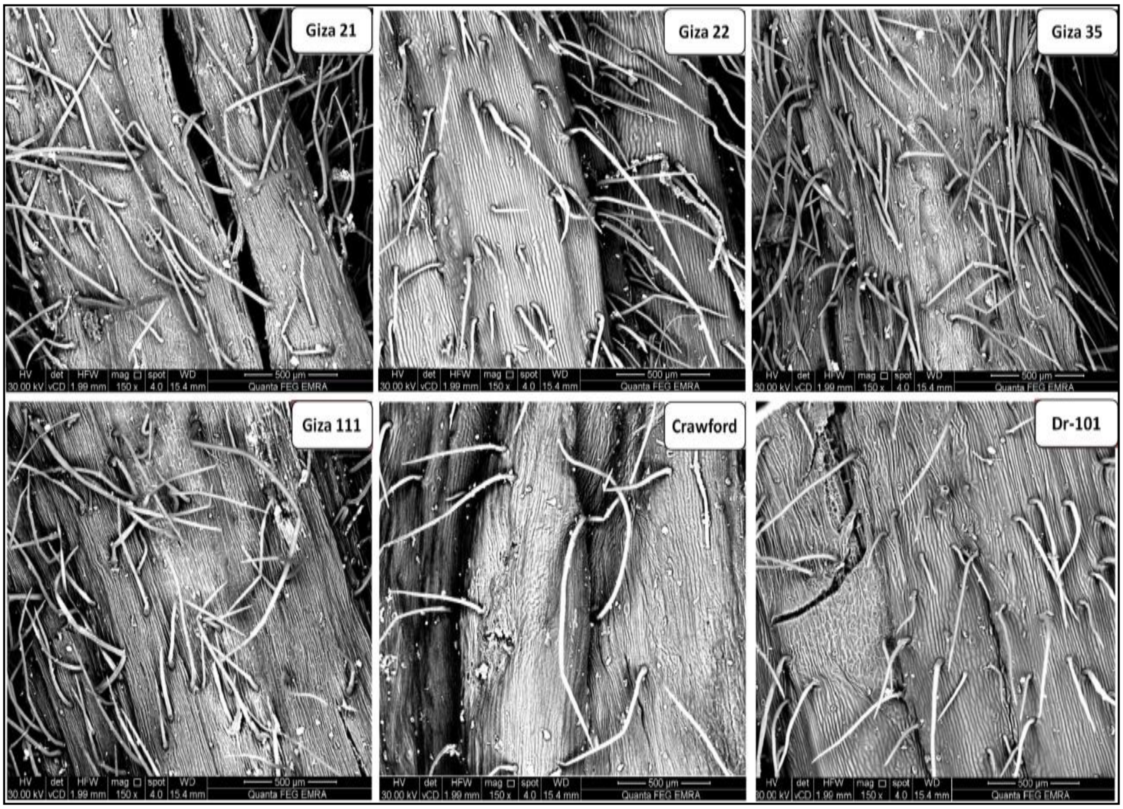

Figure S1. Different types of pubescence density on stem longitudinal section of six soybean varieties at 500 µm. The varieties showed varying levels of pubescence density, with some exhibiting a higher density than others. This variation could potentially impact the plant's ability to resist soybean stem flies.

Soybean variety	Stem pubescence length (µm)	Number of stem pubescence /500 µm	Stem pubescence density
Giza 21	526	184	dense
Giza 22	504	72	normal
Giza 35	482	232	dense
Giza 111	512	163	dense
Crawford	595	17	sparse
Dr-101	477	36	normal
LSD 0.05	68.08	49.16	---

Table S2. Different types of pubescence density on stem longitudinal section of six soybean varieties at 500 µm. The different soybean varieties exhibited variations in stem pubescence length, number, and density, reflecting genetic diversity in pubescence traits. This diversity may influence the resistance of plants to soybean stem flies.

Planting date	Soybean variety	Stem anthocyanins content (mg / 100 g FW)	Leaf total phenols content (mg/100 g DW)
mid-May	Giza 21	0.096	0.255
	Giza 22	0.087	0.230
	Giza 35	0.095	0.280
	Giza 111	0.083	0.245
	Crawford	0.077	0.220
	Dr-101	0.084	0.240
Mean		0.087	0.245
beginning of June	Giza 21	0.115	0.300
	Giza 22	0.104	0.275
	Giza 35	0.111	0.315
	Giza 111	0.099	0.290
	Crawford	0.089	0.265
	Dr-101	0.091	0.275
Mean		0.101	0.286
mid-June	Giza 21	0.108	0.290
	Giza 22	0.098	0.260
	Giza 35	0.105	0.305
	Giza 111	0.095	0.270
	Crawford	0.086	0.245
	Dr-101	0.090	0.277
Mean		0.097	0.274
Average of soybean variety	Giza 21	0.106	0.281
	Giza 22	0.096	0.255
	Giza 35	0.103	0.300
	Giza 111	0.092	0.268
	Crawford	0.084	0.243
	Dr-101	0.088	0.264
LSD 0.05 planting dates		0.01	0.03
LSD 0.05 Soybean varieties		0.008	0.02
LSD 0.05 Interaction		0.01	0.04

Table S3. Effect of planting dates, soybean varieties, and their interactions on stem anthocyanins and leaf total phenols contents. This data can offer valuable insights into the benefits of planting specific soybean varieties at optimal dates to increase the production of beneficial compounds that help in resisting soybean stem flies. By fine-tuning these factors, farmers can increase the natural defenses of soybean plants against insect infestations, resulting in higher crop yields.

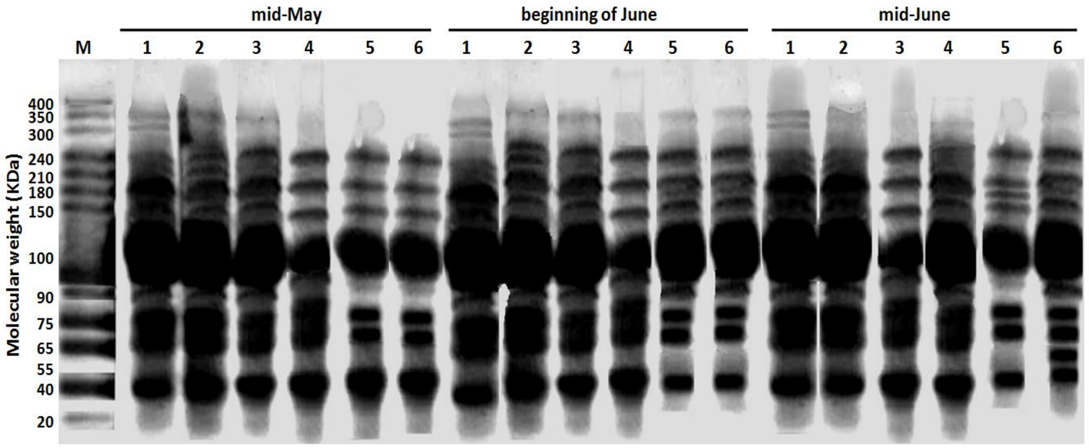

Figure S2. Banding patterns of SDS-protein PAGE for leaf of six soybean varieties through three planting dates infested by soybean stem flies. M= Marker, 1= Giza 21, 2= Giza 22, 3= Giza 35, 4= Giza 111, 5= Crawford, 6= Dr-101. The banding patterns revealed differences in protein expression levels among the soybean varieties planted on different dates. This indicates that environmental factors, such as infestation by soybean stem flies, may impact protein profiles in soybean leaves.
